# X-Ray Psoralen Activated Cancer Therapy (X-PACT)

**DOI:** 10.1371/journal.pone.0162078

**Published:** 2016-09-01

**Authors:** Mark Oldham, Paul Yoon, Zak Fathi, Wayne F. Beyer, Justus Adamson, Leihua Liu, David Alcorta, Wenle Xia, Takuya Osada, Congxiao Liu, Xiao Y. Yang, Rebecca D. Dodd, James E. Herndon, Boyu Meng, David G. Kirsch, H. Kim Lyerly, Mark W. Dewhirst, Peter Fecci, Harold Walder, Neil L. Spector

**Affiliations:** 1 Dept. of Radiation Oncology, Duke University Medical Center, Durham, North Carolina, United States of America; 2 Immunolight LLC, Detroit, Michigan, United States of America; 3 QNS Group, LLC, Bahama, North Carolina, United States of America; 4 Department of Medicine, Duke University Medical Center, Durham, North Carolina, United States of America; 5 Department of Surgery, Duke University Medical Center, Durham, North Carolina, United States of America; 6 Department of Biostatistics and Bioinformatics, Duke University Medical Center, Durham, North Carolina, United States of America; 7 Department of Neurosurgery, Duke University Medical Center, Durham, North Carolina, United States of America; Columbia University, UNITED STATES

## Abstract

This work investigates X-PACT (X-ray Psoralen Activated Cancer Therapy): a new approach for the treatment of solid cancer. X-PACT utilizes psoralen, a potent anti-cancer therapeutic with current application to proliferative disease and extracorporeal photopheresis (ECP) of cutaneous T Cell Lymphoma. An immunogenic role for light-activated psoralen has been reported, contributing to long-term clinical responses. Psoralen therapies have to-date been limited to superficial or extracorporeal scenarios due to the requirement for psoralen activation by UVA light, which has limited penetration in tissue. X-PACT solves this challenge by activating psoralen with UV light emitted from novel non-tethered phosphors (co-incubated with psoralen) that absorb x-rays and re-radiate (phosphoresce) at UV wavelengths. The efficacy of X-PACT was evaluated in both in-vitro and in-vivo settings. In-vitro studies utilized breast (4T1), glioma (CT2A) and sarcoma (KP-B) cell lines. Cells were exposed to X-PACT treatments where the concentrations of drug (psoralen and phosphor) and radiation parameters (energy, dose, and dose rate) were varied. Efficacy was evaluated primarily using flow cell cytometry in combination with complimentary assays, and the in-vivo mouse study. In an in-vitro study, we show that X-PACT induces significant tumor cell apoptosis and cytotoxicity, unlike psoralen or phosphor alone (p<0.0001). We also show that apoptosis increases as doses of phosphor, psoralen, or radiation increase. Finally, in an in-vivo pilot study of BALBc mice with syngeneic 4T1 tumors, we show that the rate of tumor growth is slower with X-PACT than with saline or AMT + X-ray (p<0.0001). Overall these studies demonstrate a potential therapeutic effect for X-PACT, and provide a foundation and rationale for future studies. In summary, X-PACT represents a novel treatment approach in which well-tolerated low doses of x-ray radiation are delivered to a specific tumor site to generate UVA light which in-turn unleashes both short- and potentially long-term antitumor activity of photo-active therapeutics like psoralen.

## Introduction

Psoralens are naturally occurring compounds found in plants (furocoumarin family) with anti-cancer [1–4] and immunogenic [5–8] properties. They freely penetrate the phospholipid cellular bilayer membranes and intercalate into DNA between nucleic acid pyrimidines, where they are biologically inert (unless photo-activated) and ultimately excreted within 24 hours. However psoralens are photo-reactive, acquiring potent cytotoxicity after ‘activation’ by ultra-violet (UVA) light [4]. When photo-activated, psoralens form mono-adducts and di-adducts with DNA leading to marked tumor cytotoxicity and apoptosis [9–11]. Cell signaling events in response to DNA damage include up-regulation of p21^waf/Cip^ and p53 activation, with mitochondrial induced cytochrome c release and consequent cell death. Photo-activated psoralen can also induce apoptosis by blocking oncogenic receptor tyrosine kinase signaling [12], and can affect immunogenicity and photochemical modification of a range of cellular proteins in treated cells [8].

Importantly, activation of psoralen can promote a strong long-term clinical response, as observed in the treatment of cutaneous T Cell Lymphoma by extracorporeal photopheresis (ECP). In ECP, malignant CTCL cells are irradiated with ultraviolet A (UVA) light in the presence of psoralen, and then re-administered to the patient. Remarkably, complete long-term responses over many decades have been observed in a sub-set of patients, even though only a small fraction of malignant cells were treated [11, 13–15]. In addition to ECP, psoralens have also found wide clinical application through PUVA treatment of proliferative skin disorders and cancer including psoriasis, vitiligo, mycosis fungoides, and melanoma [16–19]. These results suggest the activation of a clinically efficacious long-lasting immune response.

The cytotoxic and immunogenic effects of light-activated psoralen are often attributed to psoralen mediated photoadduct DNA damage [4]. A principle mechanism underlying the long-term immunogenic clinical response likely derives from psoralen induced tumor cell cytotoxicity and uptake of the apoptotic cells by immature dendritic cells, in the presence of inflammatory cytokines [3, 6, 20, 21]. However photochemical modification of proteins and other cellular components can also impact the antigenicity and potential immunogenicity of treated cells [8]. The diversity and potency of psoralen application is further illustrated by recent success using psoralen in the development of virus vaccines [5].

Despite positive clinical results, use of psoralen has to-date been restricted to superficial or extra-corporeal applications because of the inability of UVA light to penetrate into tissue (maximum penetration depth <1mm). In this work we introduce a new approach, X-PACT (X-ray Psoralen Activated Cancer Therapy), which has potential to extend psoralen therapy to a wide range of solid tumors, at deep seated sites in the body. The key innovation in X-PACT is to combine psoralen with novel phosphor particles that absorb and down-convert x-ray energy to re-radiate as UVA light. Low x-ray doses (~1Gy) are sufficient to achieve photo-activation, greatly reducing the risks of normal tissue damage from radiation.

The scope of this work is to investigate and demonstrate the feasibility of X-PACT for achieving therapeutic cytotoxicity primarily in in-vitro studies, leading to and informing a small in-vivo pilot study to demonstrate proof of concept. We explore a variety of dosimetrically important parameters, including phosphor and psoralen concentration, x-ray energy, x-ray dose and dose-rate. We also examined several different cell lines (in-vitro) to ascertain the generalizability of the approach.

## Methods

X-PACT is a novel treatment based on photo-activation of a drug (psoralen) in-situ, using very low doses of x-rays, and is not a radiation treatment that relies upon direct radiation cell kill. A principle mode of psoralen cell kill is apoptosis, prompting the use of flow-cell cytometry assays. The flow-cytometry data was complemented with other assays (methylene blue, and ATP luminescence) and importantly also with a controlled in-vivo study. Clonogenic assays were not found to be a convenient and useful tool for x-pact investigations because of the difficulty in exposing low density plated cells to phosphors (which tend to clump). This problem does not arise in flow-cell cytometry data where adequate exposure to phosphor is achieved through higher cell density.

### Psoralen

In in-vitro studies, both commercially available UVADEX (formulated psoralen from Therakos Inc, NDC 64067-216-01) and pure 8-MOP (8-methoxypsoralen from Sigma Aldrich, CAS# 298-81-7), prepared in DMSO, were investigated as alternative formulations of psoralen agents. Prior work has shown that the number of DNA photo-adducts is a linear function of the product of 8-MOP (psoralen) concentration and light-exposure [22]. Based on prior clinical experience in ECP therapy, we evaluated UVADEX and 8-MOP concentrations in the range 10–60 μM. Drug stability in the presence of phosphors was investigated using standard UV-Vis spectroscopy and HPLC-MS. In the in-vivo study, the AMT (4′-Aminomethyl-4,5′,8-trimethylpsoralen hydrochloride) psoralen derivative was used (Sigma-Aldrich (A4330). The hydrochloride salt was dissolved in sterile water for injection at a 10x stock concentration, aliquoted into amber glass vials and stored at -20C until use. Immediately prior to injection, an individual aliquot of the stock AMT solution was thawed at room temperature and diluted 10x (final 5uM) with sterile 1x PBS into a sterile glass vial containing the required amount of phosphors. Vials were gently vortexed at high speed for approximately 30 seconds to ensure adequate mixing/dispersion of the materials. Aliquots (typically 50uL) intended for injection were drawn up in single use, sterile 1mL tuberculin syringes fitted with a 28 gauge needle. All samples were protected from light and used within 3-4hours post preparation.

#### Phosphors and x-ray stimulation of UV light

**Fig 1:**
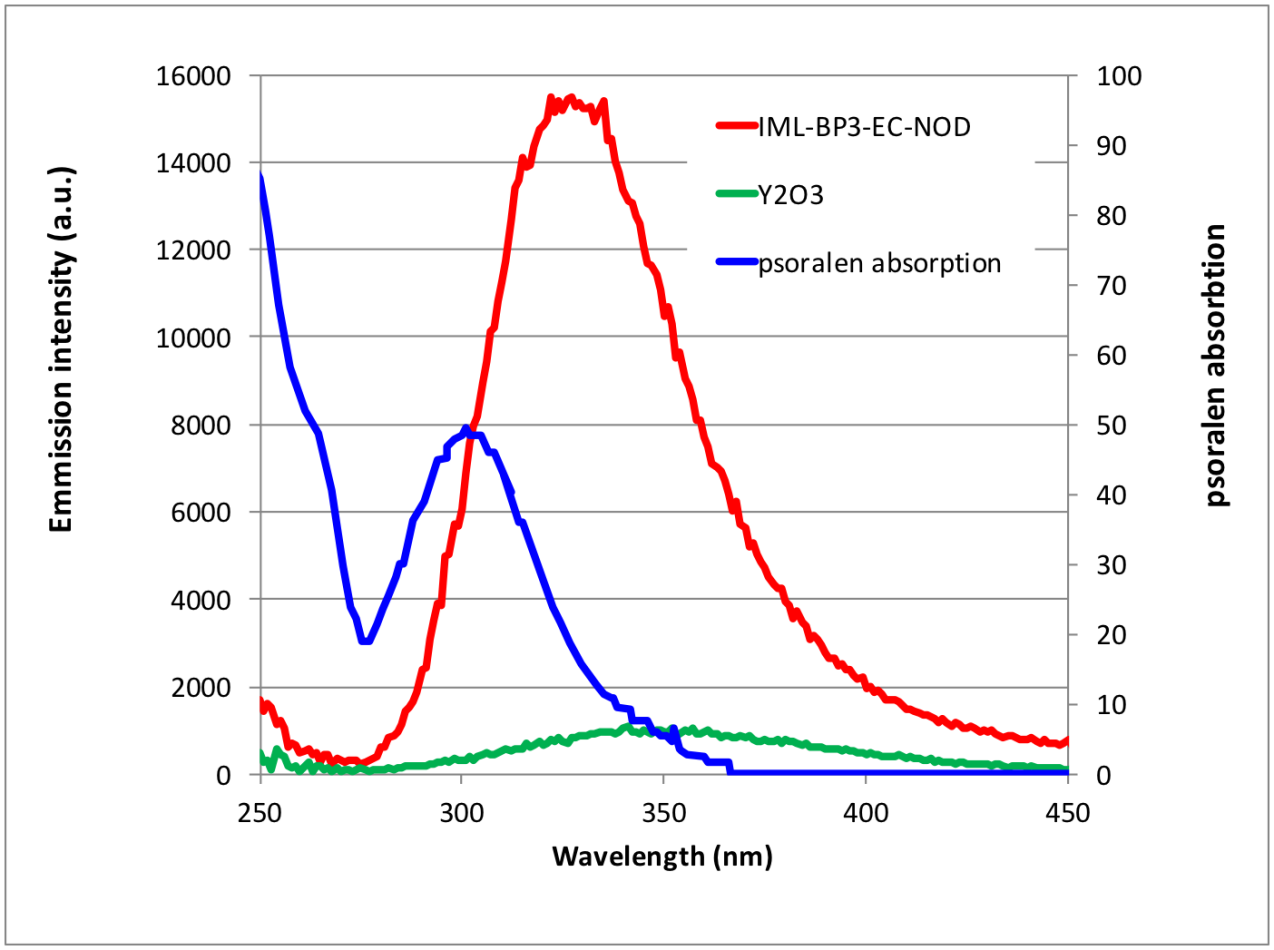
Light output from X-PACT phosphors under x-ray stimulation. The new phosphors (red line) are much brighter than previous versions evaluated in [25] (green line). The absorption spectrum of psoralen is shown for comparison (blue line).

In X-PACT therapy, psoralen is activated by light generated in-situ from phosphor particles undergoing x-ray stimulated phosphorescence. The emission profiles from the phosphor must therefore overlap the absorption/activation wavelengths of psoralen (~300–340 nm) [4]. In this study we utilize a novel combination of phosphors which were developed in house (details provided in [23, 24]), and were brighter (by a factor of ~16) than previously reported [25]. Illustrative characteristics of the phosphors are shown in Fig 1. In addition, we investigated a variety of prospective phosphor coatings with a focus on biological inertness and transparency in the UV range (maintaining the ability to activate psoralen).

### In-vitro X-PACT studies

Guava Annexin V flow cell cytometry was used to quantify cytotoxicity in 3 murine tumor cell lines (mammary -4T1; 4T1-HER2, 4T1 stably transfected with the human HER2 oncogene; glioma-CT2A; sarcoma KP-B). The mouse breast cancer cell line 4T1 was purchased from ATCC. 4T1-HER2 was kindly provided by Dr. Michael Kershaw (Cancer Immunology Program, Peter MacCallum Cancer Centre, Victoria, Australia) [26] and maintained in DMEM with penicillin/streptomycin and 10% FBS The Sarcoma KP-B cell lines were derived from primary tumors LSL-Kras; p53 Flox/Flox mice [27, 28]. Tumors between 250 and 300 cm^3^ were digested using a mixture of collagenase/dispase/trypsin for 1 hour, passed through a 70-micron filter, and cultured 5 to 8 passages before being used for experiments. Cells were cultured in DMEM medium supplemented with 10% FBS and incubated at 37°C with 5% CO2 in a humidified cell-culture incubator.

In-vitro X-PACT studies were conducted on plated cells following standard procedures. Cells were maintained in RPMI-1640 supplemented with 10% fetal bovine serum and L-glutamine from GIBCO (Grand Island, NY) growing in a humidified atmosphere of 5% CO2. After incubation, cells were trypsinized and plated evenly onto 12-well plates for 24 hours. About 20 minutes prior to X-PACT irradiation, the 12 wells of each plate were exposed to the following combinations of additives: (1) control—cells only with no additives, (2) UVADEX only, (3) phosphors only, (4) UVADEX + phosphors. Each plate had 12 wells with three wells for each of the four treatment arms. Extra duplicate plates were prepared for un-irradiated controls. The plates were then irradiated with x-rays by placing the plate at a known distance from the x–ray source (50cm, see section 2.4). After irradiation the cells were incubated on the plate for 48 hours prior to performing flow cytometry. For compatibility with 96-well Guava Nexin^®^ assay, the remaining cells were again trypsinized (after the 48 hour incubation) and plated onto the 96-well plate. Details on the Annexin V analysis are given below.

### In-vitro radiation activation technique

A range of x-ray activation protocols were investigated to determine X-PACT cytotoxic efficacy in relation to x-ray energy (kVp), total dose, and dose-rate. kV beam energies ranging between 80-100kVp were investigated. kV beams were obtained from various x-ray generating equipment, including orthovoltage units, standard diagnostic radiographic, fluoroscopic, and cone-beam computed tomography (CBCT) systems. The primary kV x-ray source utilized in this study (for all data presented, unless stated otherwise in the figure caption) was a Varian on-board-imaging x-ray source commonly found on Varian medical linear accelerators. The x-ray dose delivered for the in-vitro irradiations studied here ranged from 0.2-2Gy, with main emphasis on lower doses of 0.5-1Gy.

For x-ray irradiation, the well plates were positioned at a set distance (typically 50cm) from the x-ray source on a solid water phantom and the position of the well plates within the x-ray beam was verified by low dose kV imaging. Irradiations were typically delivered in a “radiograph” mode with kVp settings 80 and 100kVp with no added filtration in the beam (Half Value Layer = 3.0 and 3.7mm Al, respectively).

### In-vitro analysis: Quantification of Cytotoxicity and Apoptosis

Two primary flow cytometry analyses were used, both determined at 48 hours after X-PACT treatment. Cells plated in 12-well plates, where individual wells in each plate received different experimental conditions (e.g. psoralen concentration), but the same x-ray dose (i.e. all wells in a given plate receive the same x-ray dose). The first analysis evaluated was metabolic cell viability (herein referred to as **cell viability**) calculated from the number of whole cells per well as determined using forward scattering (FSC). For each well, cell viability was normalized to that in a control well without psoralen or phosphors but which did receive radiation. (All wells on a given plate receive the same dose.) The second assay is **Annexin V positivity**, which is the fraction of viable cells that are Annexin V+ by flow cell cytometry. The Annexin V (+) signal was corrected by subtracting the control signal from the no-psoralen/phosphor well on the same plate. Correcting for the control on the same plate, minimizes any potential inter-plate systematic bias associated with plating constancy or Annexin V gating parameters. The majority of plots in the results either use **cell viability** or **Annexin V(+)** staining as previously defined [29].

Other assays were used to provide independent complimentary information on cell viability, e.g. Methylene blue staining and ATP-induced Luminescence imaging (Cell-Titer-Glo^®^ Luminescence Cell Viability Assay). The luminescence imaging enabled investigation of the cytotoxicity of psoralen activated directly with a UV lamp, and in the absence of phosphors and x-ray radiation.

### In-vivo X-PACT experiments

A small trial was conducted for preliminary evaluation of X-PACT administered to syngeneic 4T1-HER2 tumors grown on BALB/c mice. The study was conducted in accordance with IACUC approved procedures within Duke University. There were 4 arms of the trial: (1) saline only (control), (2) phosphors alone with x-ray, (3) psoralen (AMT) alone with x-ray, and (4) full X-PACT treatment including both phosphor and psoralen and x-ray irradiation. 0.5Million 4T1-HER2 cells were injected subcutaneously to the right thigh of each mouse, and tumors were allowed to grow to approximately ~200 mm^3^ in volume before the initiation of the treatments. X-PACT treatments were given in 3 fractions per week, to a total of 6 fractions. In arms 2–3 a consistent x-ray irradiation technique was used (1.08Gy delivered at 75kVp by 30mA in 3 minutes) with 100μg of phosphor, and 5μM psoralen (AMT). Phosphors and/or psoralen (AMT) in 100 μl vehicle were intratumorally injected, and within 30 min after injection, x-ray irradiation was performed only to the tumor area. There were 6–8 mice per arm, and the study was repeated a second time, yielding effective sample sizes of 12–16 per arm.

### Statistical Analysis

Analysis of variance (ANOVA) was used to assess the effect of radiation (no x-ray / 1 Gy 80kVp), group (UVADEX, Phosphor, and Phosphor + UVADEX), and their interaction on two outcomes: cell viability and Annexin V (+) fraction. Linear regression examined the relationship between UV radiation exposure and cell viability for each of 3 cell lines. Multivariable linear regression was used to assess the joint effect of X-ray dose, 8-MOP psoralen dose, and phosphor dose on cell viability, and to assess the effect of 8-MOP, phosphor, and their interaction on Annexin V fraction. A two-sample t-test was used to compare 80kVp and 100kVp with respect to Annexin V fraction. A generalized linear model that used an autoregressive correlation structure to account for correlations among measurements made on the same mice was used to compare groups with respect to the rate of tumor group, as measured by the interaction between time and group. Statistical analyses were conducted using SAS 9.3 (Statistical Analysis Systems, Cary, NC).

## Results

### Characterization of high light-output Phosphors

Emission under X-Ray excitation (Fig 1) shows the new improved phosphors are 16x brighter than the tethered nano-particles (Y203) used in [25]. Phosphors can be customized so that output wavelengths match the bio-therapeutic agent to be activated. Though the phosphors are made from an inert lattice structure, they are further encapsulated by a bio compatible coating to minimize any potential reactions at the surface.

### X-PACT: In-Vitro Studies

**Fig 2:**
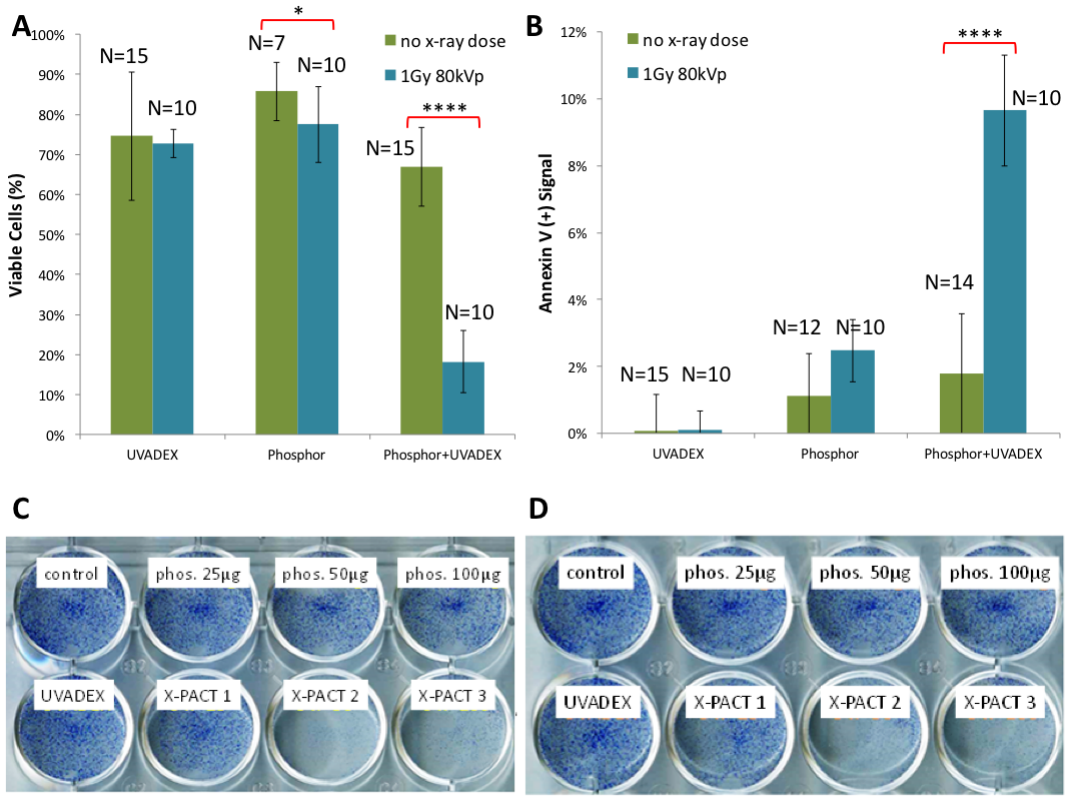
Anti-tumor effects of X-PACT and its individual components on 4T1-HER2 cells. **A:** cell viability after X-PACT (10μM 8-MOP equivalent dilution of UVADEX, 50μg/mL phosphor, 1Gy of 80kVp radiation) as determined by Guava flow cytometry. N is the number of independent measurements (different days), and error bars indicate one standard deviation. Radiation had a significantly different effect on cell viability when administered with X-PACT than with phosphor or UVADEX alone (p<0.0001 for interaction in two-way ANOVA). With X-PACT, radiation resulted in a significant decrease in cell viability relative to no x-ray dose. **B:** the Annexin V (+) fraction of viable cells shown in 3A. Radiation had a significantly different effect on Annexin V when administered with X-PACT, as opposed to individual components (p<0.0001). **C and D:** Cell viability illustrated by methyl blue staining for identical plates each receiving 1Gy of 80kVp x-rays. Each plate contained wells including no additives (control), three concentrations of phosphor only (25, 50, & 100μg/mL with DLC), UVADEX only (10uM 8-MOP equivalent dilution), and three combination X-PACT regimes.

Fig 2 illustrates the efficacy of X-PACT treatment in-vitro in 4T1-HER2 cells, utilizing an x-PACT regimen of 1/10-diluted UVADEX (with equivalent of 10μM 8-MOP), 50μg/mL phosphor 1Gy of 80kVp x-rays. Fig 2A presents the cell viability data for three treatment conditions: UVADEX alone, phosphors alone, and the X-PACT combination of UVADEX and phosphors. These data were compiled from experiments performed on 5 different days (within 1 month), including 15 separate experimental and 10 control plate irradiations. Fig 2B presents the Annexin V (+) signal for the same 3 conditions as Fig 2A. Fig 2C and 2D show corresponding images of viable cell populations revealed by methylene blue staining. Two results from two separate plates are shown, each with identical preparations to investigate reproducibility. X-PACT variants were tested corresponding to three concentrations of phosphor (25, 50, & 100μg/mL) with the UVADEX concentration fixed at 1/10 dilution (10uM 8-MOP).

### In-vitro X-PACT and other cell lines

**Fig 3:**
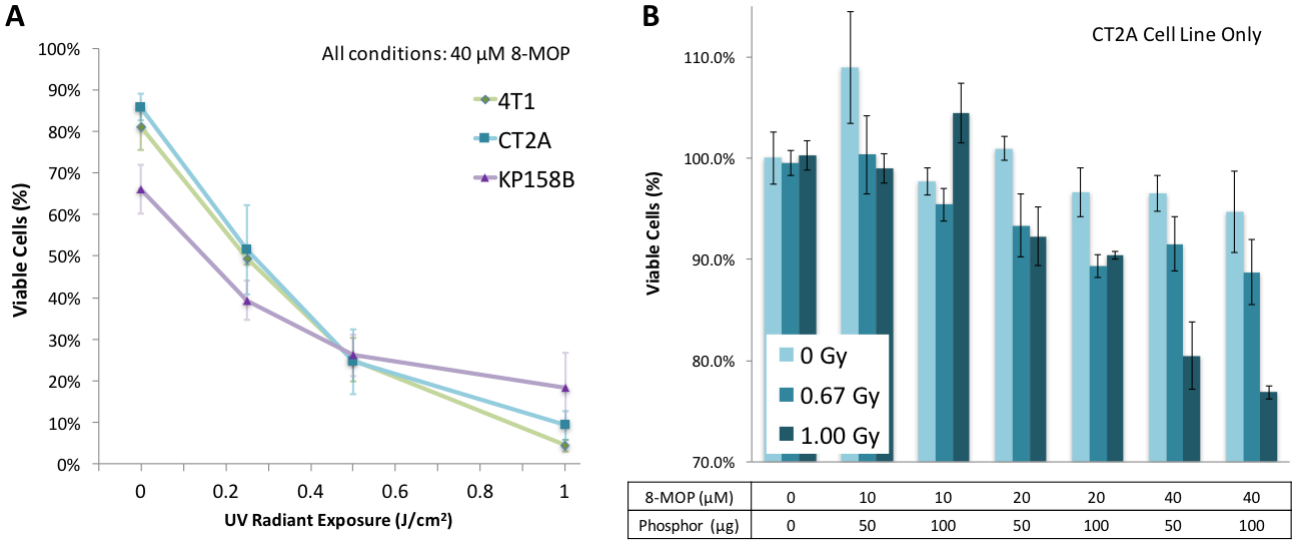
**A**: UV light activated psoralen was observed to reduce viable cells in 3 cell lines (data from Cell-Titer-Glo^®^ Luminescence Cell Viability Assay under UV light). N = 4 for each cell line at each UV light condition (0, 0.25, 0.5, 1.0 J/cm^2^). The psoralen concentration was 40 μM. Within each cell line, cell viability decreased as radiation dose increased (p<0.001; linear regression). **B:** in CT2A cells, X-PACT cytotoxicity increases with X-ray dose (0, 0.67 and 1.00 Gy respectively), concentration of 8-MOP psoralen (10, 20 and 40 μM respectively), and phosphor (50 and 100 μg/ml) respectively. Multiple linear regression showed significant reductions in cell viability with increasing radiation doses (p<0.001), increasing phosphor dose (p = 0.011), and increasing psoralen dose (p<0.001).

The relative effectiveness of UV activated psoralen on 3 independent cell lines is shown in Fig 3. Fig 3A shows sensitivity of CT2A (murine malignant glioma), 4T1 and KP-B (sarcoma) cell lines to light-activated psoralen, which is the key therapeutic mechanism of X-PACT. Fig 3B presents data on CT2A malignant glioma cells, for a range of X-PACT parameters including variable x-ray dose (0, 0.67 and 1 Gy), phosphor concentration (50 or 100 μg) and psoralen concentration (8-MOP) at 10, 20 and 40μM respectively.

### In-vitro X-PACT: Psoralen and Phosphor Concentration

**Fig 4:**
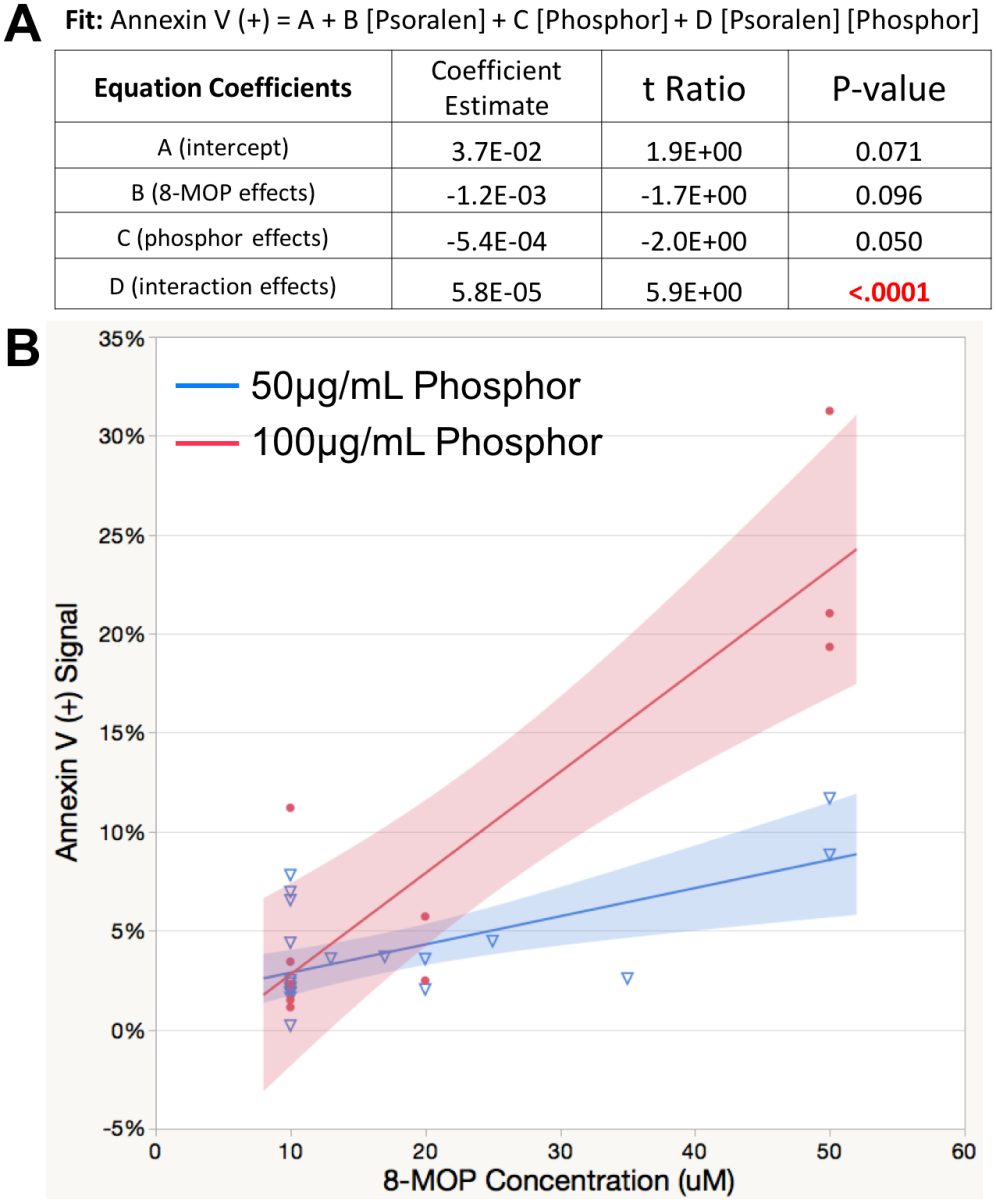
**A**: A multivariable linear regression analysis on 36 independent measurements of Annexin V (+) in 4T1-Her2 cells as a function of psoralen and phosphor concentration. All samples received an x-ray dose of 1 Gy at 80 kVp. Psoralen and phosphor concentrations ranged from 10 μM to 50 μM and from 25 μg to 200 μg respectively. The statistically significant interaction indicates that the effect of phosphor dose on Annexin levels is not consistent across levels of MOP. **B:**. graphically displays the regression model provided in 4A. The blue and red lines are the regression of psoralen on annexin when phosphor = 50 μg/mL, and 100 μg/mL respectively. The shaded regions indicate the 95% confidence limits.

Fig 4A presents a multivariable linear regression analysis on 36 independent measurements (wells) of Annexin V (+) as a function of three variables: psoralen concentration, phosphor concentration and their interaction. Psoralen and phosphor concentrations ranged from 10 μM to 50 μM and 25 μg/mL to 200 μg/mL respectively. All of the 36 X-PACT wells were irradiated with 1 Gy of x-ray radiation at 80 kVp. Data are graphically displayed in Fig 4B, along with regression lines that describe the relationship between psoralen and annexin when phosphor = 50 μg/mL (blue curve), and when phosphor = 100 μg/mL (red curve). The shaded areas represent the 95% confidence limits.

### In-vitro X-PACT: X-ray Energy and Total Dose

**Fig 5:**
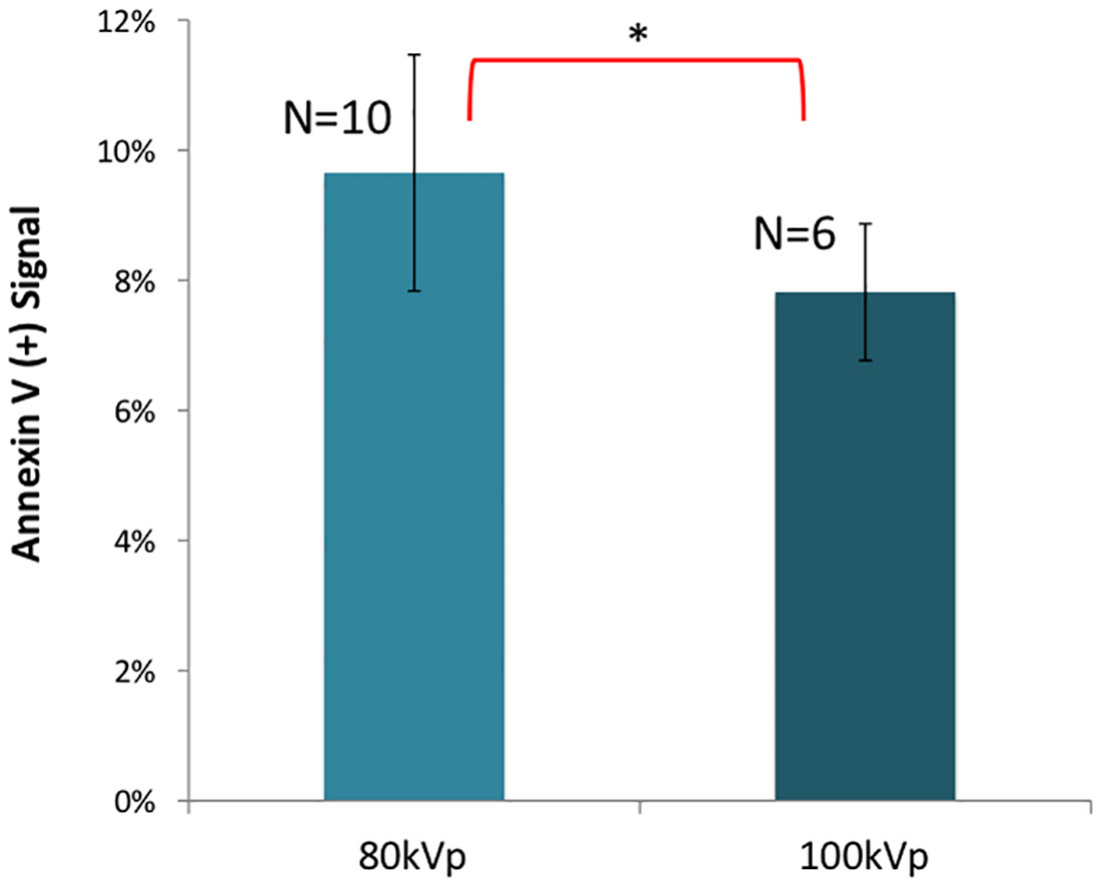
An X-PACT effect in 4T1-her2 is observed at both 80 and 100kVp, with suggestion that 80 kVp may be slightly more effective than 100 kVp (p = 0.011). This data acquired from X-PACT treatment of 4T1-HER2 cells with constant phosphor concentration of 50 μg/mL and UVADEX diluted to 8-MOP concentration of 10 μM (1:10 dilution). N is the number of independent measurements.

Fig 5 compares X-PACT at two different x-ray energies (80 and 100 kVp). These experiments involved 4T1-HER2 cells treated with 10 μM 8-MOP equivalent UVADEX, and 50 μg/mL phosphors.

### In-vivo X-Pact Experiments

**Fig 6:**
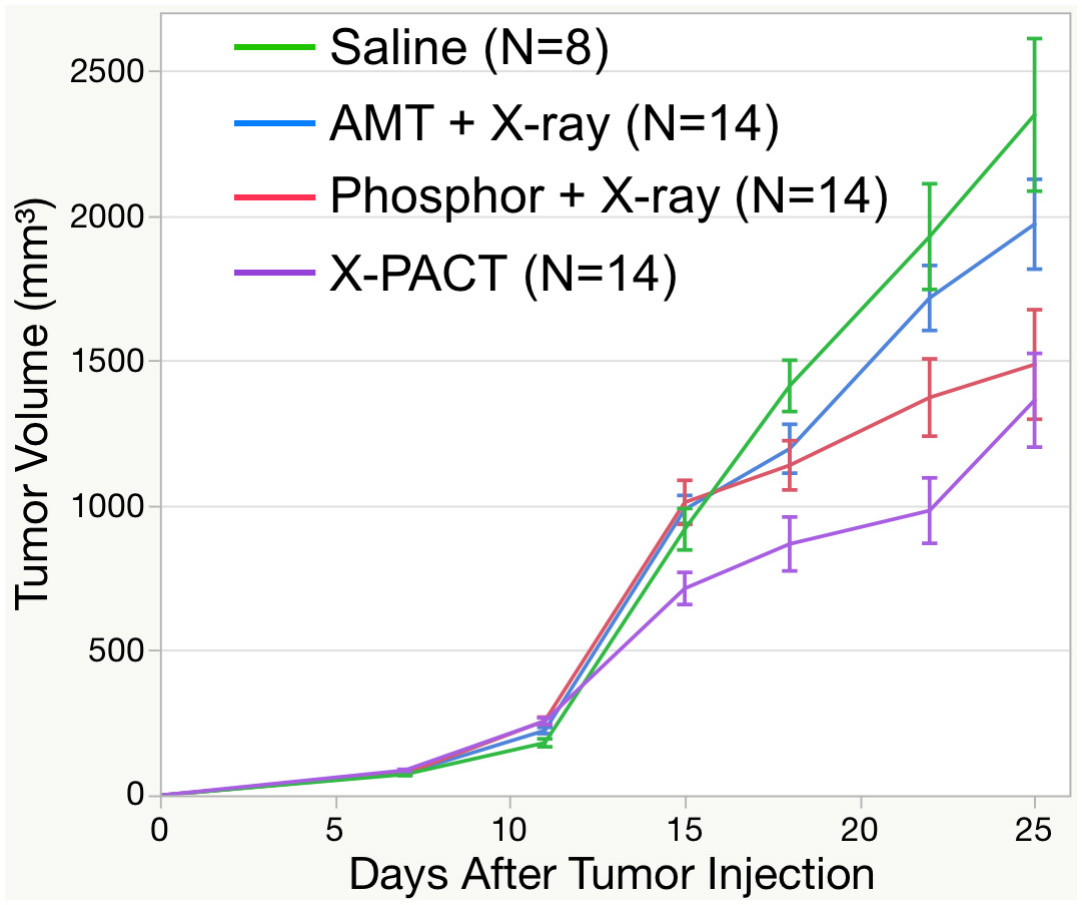
In-vivo investigation of X-PACT application to BALB/c mice with syngeneic 4T1-HER2 tumors. Error bars are 1 standard error of the mean. A generalized linear model that used an autoregressive correlation structure to account for within-mouse correlations showed the rate of tumor growth after initiation of treatment to differ significantly among the 4 groups (p<0.0001; interaction between time and group). The rate of tumor growth within the phosphor + X-ray and X-PACT groups was significantly slower than that observed in the saline (p<0.0001 and p<0.0001, respectively) and AMT + X-ray groups (p = 0.0073 and p = 0.0011, respectively).

**Fig 7:**
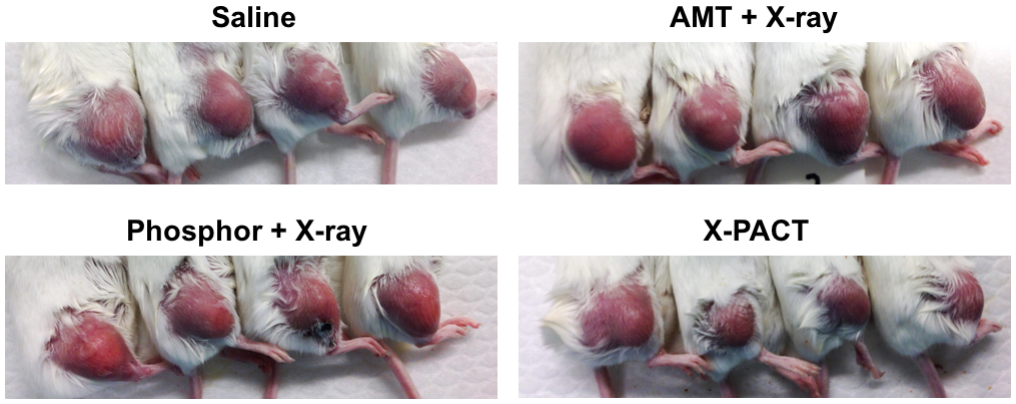
a) Representative tumor size for each arm: day 25 after tumor cell implantation.

Tumor growth curves from the in-vivo X-PACT irradiation of syngeneic 4T1-HER2 tumors are shown in Fig 6. Treatment for all 4 groups (saline, AMT + X-ray, Phosphor + X-ray, and X-PACT) was initiated on day 11 after tumor cell implantation. A consistent x-ray irradiation technique was used for all arms (except saline control) which was 1.08Gy delivered at 75kVp by 30mA in 3 minutes. Mice tolerated well for the treatments, and no significant side effects were observed (Fig 7). A comparison of the rate of tumor growth after initiation of treatment within the 4 groups showed a significant difference (p<0.0001; interaction between day and group). The rate of tumor growth within the phosphor + X-ray and X-PACT groups was significantly slower than that observed in the saline (p<0.0001 and p<0.0001, respectively) and AMT + X-ray groups (p = 0.0073 and p = 0.0011, respectively).

## Discussion

In X-PACT therapy, psoralen is activated by light generated in-situ from phosphor particles undergoing x-ray stimulated phosphorescence. The emission profiles from the phosphor must therefore overlap the absorption/activation wavelengths of psoralen. Previously, nano-scintillating particles were developed which were tethered to psoralen [25]. This approach led to reduced psoralen intercalation due to the presence of the tethered molecule. In this work we simplified the system by eliminating the tethering process and replacing it by a co-incubation of psoralen and phosphor particles. The free psoralen benefits from a high degree of mobility and greater intercalation with DNA. Another key benefit to eliminating tethering is the opportunity to utilize novel bright phosphors of different particle size and distribution which can be customized for specific absorption and emission spectra.

In the 4T1 in-vitro cell viability analysis (Fig 2A), we show that radiation has a significantly different effect on cell viability when administered with X-PACT than with phosphor or UVADEX alone (p<0.0001). With phosphor and psoralen present, the administration of x-ray resulted in a decrease in viable cells from 70 to 20%. The effect of adding radiation to the control conditions (phosphor alone or psoralen alone) did not lead to a substantial reduction in cell viability. The increased toxicity associated with the presence of both phosphors and x-rays could be attributed to DNA damage arising by UV light from x-ray induced phosphorescence from the phosphors. Substantial cytotoxicity (~80%) was only observed in the full X-PACT arm demonstrating the synergistic therapeutic effect of the combination of phosphor, UVADEX and radiation.

In the 4T1 in-vitro apoptotic analysis (Fig 2B), cells exposed to UVADEX alone (left bars) exhibited negligible apoptotic activity either with or without x-ray (p values of 0.90 and 0.09 respectively). There was a slight increase in Annexin V staining when cells were exposed to phosphor alone (middle bars) (~1%, p = 0.098) suggesting a slight toxicity of the phosphors. However, it was only when both phosphor and UVADEX were combined (right bars) that a statistically significant increase in Annexin V staining was observed (~8%, p<0.0001), indicating an increase in apoptosis. The anti-tumor effects of X-PACT were further illustrated in the methyl blue staining in Fig 2C and 2D. In both the X-PACT 2 and 3 conditions, little effect was observed for the individual components of UVADEX and phosphor. The methyl blue staining results are consistent with the flow cytometry data, in that all X-PACT components are required for high cytotoxicity. Less cytotoxicity is manifest in the first X-PACT condition because of decreased phosphor concentration.

When X-PACT and components were evaluated (Fig 3A), a regression analysis shows that an increase in radiation dose results in a significant decrease in cell viability (p<0.001) within each cell line. This observation suggests that X-PACT may have applicability to a range of different tumor types. In CT2A malignant glioma cells, X-PACT cell cytotoxicity was observed (Fig 3B) to increase with the magnitude of X-ray dose (0, 0.66 and 1Gy respectively), concentration of 8-MOP psoralen (10, 20 and 40 μM respectively), and phosphor (50 and 100 μg/ml respectively). A linear model assessed the joint effect of psoralen dose, phosphor dose, and radiation dose on cell viability and showed that significant reductions in cell viability occurred with increasing radiation doses (p<0.001), increasing phosphor dose (p = 0.011), and increasing psoralen dose (p<0.001).

In the most comprehensive in-vitro 4T1 analysis (Fig 4A) a model was generated that explained 72% of the variability associated with the Annexin outcome as a function of 8-MOP dose, phosphor dose, or their interaction. The statistically significant interaction (p<0.0001) is an indicator of an enhanced effect when phosphor and psoralen were present. The magnitude of effects are graphically displayed in Fig 4B. A general observation from this data, acquired with constant x-ray dose, is that apoptotic fraction induced by X-PACT increases with either increasing phosphor or psoralen concentration.

The final in-vitro study investigated whether changing x-ray energy had much effect on X-PACT efficacy (Fig 5). Phosphor design considerations indicated that ~80kVp would be optimal, but a higher energy would have an advantage from treatment delivery perspective (greater penetration in tissue). For this reason 100kVp beam energy was investigated. An increase in apoptotic signal (over the control) was observed for X-PACT treatments at both energies. The data suggests the possibility of a slightly greater effect at 80 kVp.

X-PACT therapy seeks to engage the anti-tumor properties of psoralens activated in-situ, in solid tumors, with the potential for engaging a long-term response [4]. The data presented in Fig 6, show the first in-vivo application. The first X-PACT treatment was delivered to the syngeneic 4T1-HER2 tumors, on day 10 after implantation. Over the next two weeks a growth delay was observed in the X-PACT treatment arm. The statistical analysis showed 1) The rate of tumor growth in the saline and AMT + X-ray group are not significantly different. 2) The rate of tumor growth in the Phosphor + X-ray group is significantly slower than the growth in the saline or AMT + X-ray group. 3) The rate of tumor growth in the X-PACT group is significantly slower than the growth in the saline and AMT + X-ray groups. 4) The rate of tumor growth in the Phosphor + X-ray group and the X-PACT group were suggestive, but not significantly different.

Taken together, the in-vitro and in-vivo data presented here demonstrate basic efficacy for X-PACT, and present a foundation and rationale for future studies to investigate in further detail. Future studies are required to further optimize all aspects of the technique, including phosphor efficiency (both to x-ray activation energy and UV light output), psoralen dosing and x-ray irradiation technique. Future studies will also evaluate host response, including potential for stimulation of immune response.

## Conclusions

Medical applications of ionizing radiation have traditionally involved diagnostic imaging and radiation therapy. Diagnostic imaging (planar x-rays and x-ray-CT) utilizes low energy x-rays, in order to obtain better soft-tissue—bone contrast, and lower dose exposure to the patient. In radiation therapy, higher energy MV radiation (6MV and higher) is typically used to achieve skin sparing. The X-PACT therapeutic paradigm departs from these conventions by utilizing low energy radiation (and low doses) to initiate phosphorescence of UV light in-situ, in potentially deep seated lesions, for the purpose of activating a potent anti-tumor photo-bio-therapeutic (psoralen). This work presents in-vitro investigations that demonstrate the basic viability of this approach, confirming x-ray activation of psoralen. A novel finding is the demonstration of the ability to obtain a measurable anti-tumor response without the need for complicated tethering of the psoralen and phosphor particle. This simplification enables utilization of specially designed super-bright particles (Fig 1) and may facilitate migration to clinical application. Further work will investigate the potential for treatment optimization and efficacy through a compassionate use clinical study in canine patients (spontaneous tumors). The longer term potential is the possibility of precise local activation of psoralen in-situ at any depth, and stimulation of an anti-tumor immune response.
